# A279 EVIDENCE OF TRANSIENT RECEPTOR POTENTIAL MELASTATIN 3 (TRPM3) CHANNEL SENSITIZATION IN A MOUSE MODEL OF COLITIS

**DOI:** 10.1093/jcag/gwac036.279

**Published:** 2023-03-07

**Authors:** J W King, A S W Bennett, H Wood, C Baker, D E Reed, A E Lomax

**Affiliations:** 1 Department of Medicine; 2 Department of Biomedical and Molecular Sciences, Queen's University, Kingston, Canada

## Abstract

**Background:**

Abdominal pain is a primary symptom of inflammatory bowel disease (IBD). Opioids provide relief from IBD-associated pain, but they are addictive and associated with excess mortality in IBD patients. Thus, there is a need to develop novel therapeutics for IBD-associated pain. The mechanosensitive ion channel, transient receptor potential melastatin 3 (TRPM3) is upregulated in sensory neurons innervating inflamed tissue and contributes to pain from inflamed joints and cystitis. However, TRPM3’s role in abdominal pain has not been investigated.

**Purpose:**

To evaluate whether TRPM3 contributes to abdominal pain using a mouse model of colitis.

**Method:**

We used ratiometric Ca^2+^ imaging and extracellular afferent nerve recording to determine the effects of pharmacological activation or inhibition of TRPM3 on T13-L5 dorsal root ganglia (DRG) neurons and lumbar splanchnic nerves, respectively. Increased intracellular Ca^2+^ indicates neuronal excitation. Furthermore, the effects of TRPM3 activation in neurons and nerves from healthy mice and mice with dextran sulphate sodium-induced colitis were compared.

**Result(s):**

The TRPM3 agonists, CIM-0216 (0.1-10µM) and pregnenolone sulphate sodium (PSS; 1-300µM), concentration-dependently increased intracellular Ca^2+^ concentration in mouse DRG neurons and this was blocked using the TRPM3 inhibitor isosakuranetin (5µM; p<0.0001, Mann-Whitney Test). CIM-0216 (5µM)-induced increases in intracellular Ca^2+^ were significantly larger in neurons from mice with colitis (326±16% of baseline) compared to neurons from healthy mice (257±13% of baseline; p<0.01, Kruskal-Wallis with Dunn’s Multiple Comparison Test). The percentage of neurons responding to CIM-0216 was significantly increased in mice with colitis compared to healthy mice (79% vs 62%; p<0.001, Fischer’s Exact Test). Similarly, the percentage of neurons responding to PSS from mice with colitis was increased compared to healthy mice; however, this did not reach statistical significance (75% vs 70%, p=0.351, Fischer’s Exact Test). Furthermore, CIM-0216 (20µM)-induced change in the basal firing of lumbar splanchnic nerves was significantly increased in mice with colitis (1.23±0.24Hz) compared to healthy mice (0.60±0.14Hz, p<0.05, unpaired t-test).

**Conclusion(s):**

TRPM3 activation excited DRG neurons and lumbar splanchnic nerves and these excitatory effects were augmented in mice with colitis.

**Disclosure of Interest:**

None Declared

